# Bone Regeneration in SLS-Manufactured Resorbable 3D-Scaffolds—An Experimental Pilot Study in Minipigs

**DOI:** 10.3390/polym17182498

**Published:** 2025-09-16

**Authors:** Philipp Kauffmann, Susanne Wolfer, Tim Gellhaus, Christina Behrens, Christian Dullin, Frank Reinauer, Tobias Wolfram, Stefanie Grom, Marijan Vučak, Sabrina Hauspurg, Claudia Rode, Ralf Wyrwa, Henning Schliephake

**Affiliations:** 1 Department for Maxillofacial Surgery, Universitätsmedizin Goettingen, Robert-Koch-Str. 40, 37075 Göttingen, Germany; philipp.kauffmann@med.uni-goettingen.de (P.K.); susanne.wolfer@med.uni-goettingen.de (S.W.); tim.gellhaus@gmail.com (T.G.); cbehren@gwdg.de (C.B.); 2 Department of Diagnostic and Interventional Radiology, George-Augusta-University, Robert-Koch-Str. 40, 37075 Göttingen, Germany; christian.dullin@med.uni-goettingen.de; 3 Department of Diagnostic and Interventional Radiology, University Hospital Heidelberg, 69120 Heidelberg, Germany; 4 Max Plank Institute for Multidisciplinary Sciences, 37075 Göttingen, Germany; 5 KLS Martin SE & Co. KG, 78570 Mühlheim, Germany; frank.reinauer@klsmartin.com (F.R.); tobias.wolfram@klsmartin.com (T.W.); stefanie.grom@klsmartin.com (S.G.); 6 Schaefer Kalk GmbH & Co. KG, 65582 Diez, Germany; marijan.vucak@schaeferkalk.de; 7 INNOVENT e.V., Prüssingstr. 27B, 07745 Jena, Germany; s.hauspurg@innovent-jena.de; 8 BLINK AG, 07747 Jena, Germany; claudia_r@blink-dx.com; 9 Fraunhofer-Institute for Ceramic Technology and Systems IKTS, Michael-Faraday-Straße 1, 07629 Hermsdorf, Germany; ralf.wyrwa@ikts.fraunhofer.de

**Keywords:** poly-lactic acid, scaffold, CAD/CAM, selective laser sintering

## Abstract

**Background**: The aim of this experimental pilot study was to evaluate the effect of pore volume and material composition on bone ingrowth into a resorbable poly-L-lactide-CaCO_3_/CaP scaffold. **Methods**: Cylindric scaffolds of 7 mm diameter and 5 mm height and two different degrees of porosity were produced using selective laser sintering of poly-L-lactide-powder containing 24% CaCO_3_ spherulites with and without surface modification with 4% CaP. Six minipigs received the four types of macroporous cylindrical scaffolds, inserted press fit into trephine defects of the tibial metaphyses, and left to heal for 4 and 13 weeks in three animals each. The specimens were evaluated using µCT for pore volume fill, and histomorphometry for bone formation and immunohistochemistry for expression of osteocalcin. **Results**: After *4 weeks*, newly formed bone ranged from 2.73 mm^2^ to 5.28 mm^2^ mean total area. Mean pore volume fill varied between 12.25% and 20.35% and the average level of osteocalcin expression ranged from 2.49 mm^2^ to 4.48 mm^2^ mean total area. No significant differences were found between the different scaffolds. After *13 weeks,* bone formation and pore fill volume had significantly increased in all scaffold groups up to a mean value of 14.79 mm^2^ and 96.04%, respectively. Again, differences between the groups were not significant. **Conclusions**: The tested SLS produced scaffolds allowed for bone ingrowth, almost completely filling the pore volume after 13 weeks. Newly formed bone was in direct contact with the scaffold walls. Differences in pore volume did not account for significant differences in bone formation inside the scaffolds. The addition of CaP likewise did not lead to increased bone formation, most likely due to low availability of CaP to the biological environment.

## 1. Introduction

The use of block-shaped bone grafts for three-dimensionally stable alveolar ridge augmentations remains a challenge when non-autogenous grafting material is considered. In particular, xenogenic and synthetic block grafts are difficult to use, as the brittle material characteristics often impair precise adaptation and fixation to the underlying bone. In this respect, the use of 3D data of the individual defect anatomy for additive manufacturing of 3D scaffolds constitutes a promising approach to the production of individually preshaped bone graft blocks for alveolar bone repair. The major advantages of this technique are not only the ability to adjust the shape of the scaffolds to the individual anatomy of bone defects but also the possibility to vary material composition according to the individual requirements of the recipient bed [1,2]. A frequently employed process for the manufacturing of 3D scaffolds is selective laser melting (SLM) or selective laser sintering (SLS), where a 3D object is printed layer by layer through a laser beam in a powder bed [3,4]. Selective laser melting uses temperatures at which the powder is completely melted and is preferably employed for the production of patient-specific metallic implants. Selective laser sintering uses temperatures just below the melting point of the material, which results in molecular fusion on the surface of the powder particles. This leads to sintering of the powder with definition of the 3D shape without total melting of the material [4]. This approach is particularly suitable for printing of resorbable polymer scaffolds [5,6,7,8].

The most frequently used degradable polymers in medicine are composed of poly-alpha-hydroxy acids such as poly-lactic acid (PLA), poly-glycolic acid (PGA), poly-caprolactone (PCL) [9] or poly-dioxanone (PDA) [10]. Particularly, the use of PLA and PGA is associated with the occurrence of acidic degradation products that can lead to foreign body reactions and inhibit bone contact with the surface of resorbable polymer PLA scaffolds by formation of an intervening soft tissue layer [11,12,13]. The decrease in periimplant pH associated with these acidic degradation products can be avoided by the addition of basic CaCO_3_ or CaP particles during scaffold production. This has been shown to lead to a near constant physiological pH in vitro during the entire process of degradation of resorbable PDLLA scaffolds produced through supercritical gas foaming of poly-DL-lactic acid-ACP-CaCO_3_-powder [14]. In vivo, addition of basic particles such as hydroxylapatite has been associated with direct contact between bone and the polymer composite [15,16]. The use of SLS for the production of resorbable scaffolds from poly-lactic acid- CaCO_3_ powder would allow for the creation of degradable block-shaped bone grafts with a well-controlled open porosity in the µm range, which can allow for bone ingrowth and at the same time avoid untoward effects of polymer degradation on bone formation. The feasibility of this approach on the material side has already been shown in in vitro studies [17]. The present experimental pilot study was now conducted to move on to the next step of evaluation to make sure that the material does exert the intended function (i.e., allow for bone regeneration) without causing adverse tissue reactions. This requires the transition from the in vitro environment to the in vivo setting. The current study thus evaluates the in vivo performance of additively manufactured 3D resorbable polymer scaffolds using SLS from PLA-CaCO_3_/CaP powder with different pore volumes and compositions. The necessary preparatory work for material characterization and biocompatibility testing was carried out at Innovent. The aim of the study was to test the hypothesis that modifications in scaffold architecture and material composition have an effect on bone ingrowth into the macroporous resorbable scaffolds.

## 2. Materials and Methods

The minipig was chosen as an experimental animal for the in vivo testing for three reasons: (i) the anatomy allows for the testing of clinically relevant scaffold volumes, (ii) the species is well established in preclinical research on implants and bone regeneration, (iii) bone physiology and bone turnover are comparable to humans [18]. For the present pilot study on material characteristics, the tibia diaphysis was chosen for implant placement to avoid complications with wound dehiscences and subsequent infections frequently encountered with transoral procedures in ridge defects in this species [19]. Four types of implants were planned (see below), and two intervals were scheduled for evaluation (4 and 13 weeks).

### 2.1. Sample Size Calculation

The number of animals needed was calculated according to Mead’s resource equation **E = N − B − T** [20]. This equation is designed to save resources in complex biological experiments with multiple treatments. **N** is the total number of experimental units (defects), **B** is the number of blocking effects (environmental conditions, housing, or grouped treatment), and **T** is the number of treatment groups. If the number of experimental units **N** is adequate, **E** should have a value between 10 and 20 [21].

In the experimental design, four defects were planned per animal in the tibiae on one side. Hence, the number of blocking effects **B** (grouped treatment) would be B = N/4. The experiments involved four treatments (two variations in scaffold architecture and two variations in material composition). With results being assessed at 2 intervals (after 4 and 13 weeks), a factorial design of 4 treatments × 2 intervals resulted in 8 groups. With a maximum value of **E** = 20, the maximum number of experimental units N (defects) required is calculated by 20 = N − N/4 − 8. resulting in N = 37.3 defects, which was equivalent to 9.3 animals. Hence, the number of animals used was *n* = 10, with 5 animals each being assigned to the two intervals.

### 2.2. Scaffold Production

**Figure 1:**
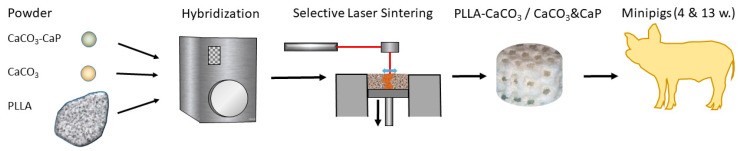
Outline of the experimental setting: Hybridization of CaCO3/CaCO_3_&CaP and PLLA, scaffold production using SLS, implantation into minipigs for 4 weeks and 13 weeks.

The scaffolds were produced through selective laser sintering of a composite powder of poly-L-lactide combined with CaCO_3_, with and without surface modification with CaP. The addition of CaP was intended to improve the bioactivity of the scaffold with respect to bone formation. The composite powder had been produced and characterized by Schaefer Kalk as previously described [17]. In brief, poly-L-lactic acid (PLLA) (Resomer L206 S, Evonik, Essen, Germany, inherent viscosity range 0.8–1.2 gL/g) granules and spherulite-shaped precipitated calcium carbonate—with and without CaP (calcium phosphate coating (4%)—were dry processed in an impact mill (NHS-1, Tokyo 143, Japan) at 6400 rpm, leading to an inclusion of the spherulites into the PLLA granules. The choice of materials and composition was based on previous experiments [17]. Poly-L-lactide with an inherent viscosity of 1 dL/g was used, as this has shown the best processability by SLS. The volume fraction of the CaCO_3_-/CaCO_3_-CaP spherulites in the composite particles was approximately 24%. The CaCO_3_ filler content was chosen based on preliminary SLS experiments and represents a compromise between medical demand (preferably high filler content) and processability by SLS (preferably low filler content). The amount of phosphate added had been determined during additional experiments that had shown that the addition of 4% CaP had resulted in complete coverage of the CaCO_3_-spherulites with CaP. The median particle size of the resulting composite particles ranged between 55 and 57 μm. The experimental design is outlined in Figure 1

**Figure 2:**
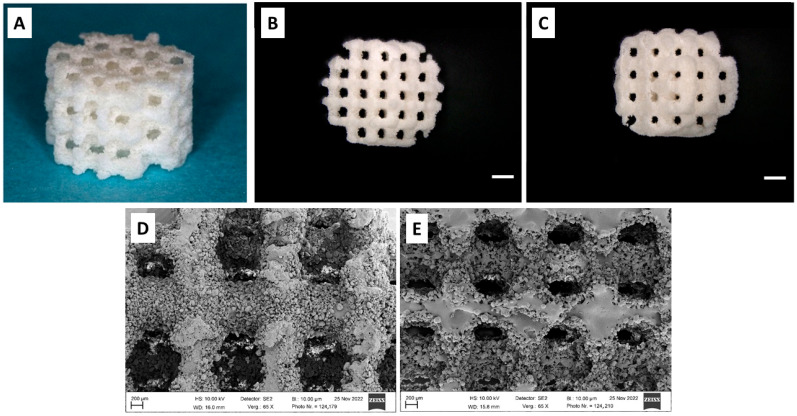
(**A**): cylindrical scaffold of 7 mm diameter and 5 mm height; (**B**): scaffold with 800 µm scaffold web thickness, bar: 1 mm; (**C**): scaffold with 1000 µm scaffold web thickness, bar: 1 mm; (**D**): PLLA-CaCO_3_ scaffold, bar: 200 µm; (**E**): PLLA-CaCO_3_/CaP scaffold, a large number of unmelted polymer composite granules is attached to the scaffold surface as residuals after laser sintering, bar: 200 µm.

The scaffolds were designed and produced by KLS Martin on an SLS machine (Formiga P110 Velocis, EOS GmbH, Krailling/München, Germany, focus diameter: 220 µm, 2.2 W, 1200 mm/s) in cylindrical shape of 7 mm diameter and 5 mm height, resulting in a scaffold volume of 192 mm^3^ (Figure 2A). The scaffold architecture was varied in thickness of scaffold webs between pores (1000 µm and 800 µm) with a constant pore size of 400 µm, resulting in two different pore volumes (69 m^3^ and 79 mm^3^, equivalent to a porosity of 35.9% and 41.1%, respectively). In conjunction with the two different types of composite powder, four types of scaffolds were produced (Figure 2B–E):

Group (1) PLLA-CaCO_3_ scaffolds with 1000 µm scaffold web thickness (pore volume 69 mm^3^/35.9% porosity).

Group (2) PLLA-CaCO_3_ scaffolds with 800 µm scaffold web thickness, (pore volume 79 mm^3^/41.1% porosity).

Group (3) PLLA-CaCO_3_/CaP scaffolds with 1000 µm scaffold web thickness, (pore volume 69 mm^3^/35.9% porosity).

Group (4) PLLA-CaCO_3_/CaP scaffolds with 800 µm scaffold web thickness, (pore volume 79 mm^3^/41.1% porosity).

### 2.3. Surgical Procedures

All surgical procedures, housing, and animal care were carried out in accordance with the German legislation for animal protection and the regulations for animal experiments of the state of Lower Saxony. The trials were reported and admitted under the license number 33.19-42502-04-21/3774 of the Office for Consumer Protection and Food Safety of Lower Saxony (LAVES). Surgical experiments were only permitted to be performed on one leg per animal. Animals were held in groups of 2–3 animals in cages with a concrete floor with saw dust bedding, and wooden walls. They were allowed to accommodate for four weeks prior to the beginning of the clinical procedures. All the animals were presented in good health. All the surgical procedures were conducted in the animal facilities of the University Medicine Goettingen following the ARRIVE guidelines [22].

The experiments were conducted between 07/2022 and 12/2022. Qualified veterinarians performed the sedation and general anesthesia and the postoperative care. Sedation was initiated by oral administration of 0.5 mg/kg body weight of Diazepam, followed by intramuscular injection of 7.5 mg/kg body weight Ketamine and 0.375 mg/kg body weight Midazolam i.m. approximately 30 min later. General anesthesia was induced with titrated i.v. administration of 1–2 mg/kg Propofol and 10 µg/kg Fentanyl, followed by endotracheal intubation. Anesthesia was maintained using Propofol (5–8 mg/kg/h) and Fentanyl (10–15 µg/kg/h). A Dexpanthenole lotion (Bepanthen**©**, Bayer AG, 51368 Leverkusen, Germany) was used to cover the eyes. Vital parameters were monitored using ECG, pulse oximetry, end-tidal CO_2_ measurement, and rectal body temperature.

**Figure 3:**
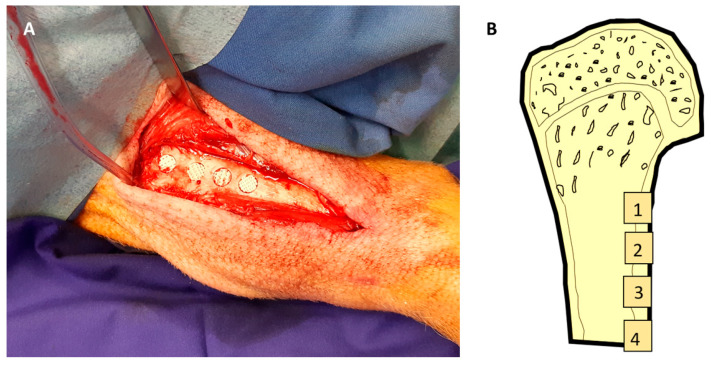
(**A**): clinical picture of implant placement; (**B**): schematic illustration of implant locations in the minipig tibia.

The tibia on one side was exposed subperiosteally from an anterior incision. The side of each animal was assigned randomly through drawing lots. Cylindrical cavities of 7 mm diameter and 5 mm depth were created in the diaphysis in a linear fashion, approx. 1 cm apart (Figure 3A,B). The implants were inserted press fit into the defects. Wound closure was performed in layers using resorbable sutures (Vicryl 3.0, Ethicon, Norderstedt, Germany). The position of the individual scaffolds was allocated randomly through drawing lots.

During the immediate postoperative period (1 week), animals were visited twice per day. For reduction in postoperative pain, 5 mg/kg body weight Carprofen was administered orally daily during the first postoperative week. When animals showed signs of discomfort, 50 mg/kg body weight Metamizol was administered orally.

During the postoperative period, tibial fractures occurred in 4 out of the 10 animals, resulting in 6 animals remaining in the experiment. As the number of 24 defects that were available in these 6 animals was equivalent to the minimum number of animals required according to Mead’s equation used for sample size calculation, it was concluded to continue with the reduced number of animals.

### 2.4. Outcome Parameter

The tibia of 3 animals each were removed after 4 weeks and 13 weeks, with retrieval of scaffolds together with adjacent bone using a diamond saw (EXAKT**©**, Robert-Koch-Str. 5, 22851 Norderstedt, Germany). The following outcome parameters were defined:

- Primary outcome parameters:

Bone formation and pore volume fill assessed through µCT (mm^3^);

Bone formation assessed through histomorphometry (mm^2^);

Osteocalcin expression assessed through immunohistochemistry (mm^2^).

Osteocalcin has been selected for assessment of osteogenic activity of the ingrowing soft tissue as it is considered as a late marker of osteogenic differentiation indicating terminal osteogenic differentiation of mesenchymal [23,24] and has been used in previous experiments on the use of polymer scaffolds in bone regeneration [25].

### 2.5. Micro-CT, Histologic Preparation, and Morphometry

**Figure 4:**
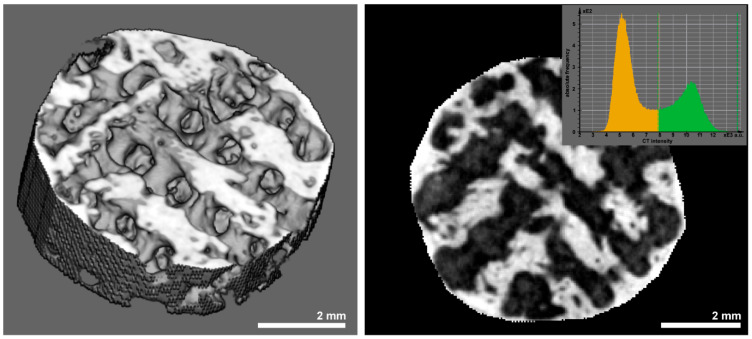
Display of 3D structure of a scaffold (**left** side) and cross-section through a scaffold (**right** side). Illustration of the identification of the scaffold material (density values of >7300 arbitrary units/green area in the histogram in the inlet) and bone tissue (density values < 7300 arbitrary units/yellow area in the histogram).

Before preparation for histologic and morphometric evaluation, the scaffolds were subjected to analysis in a µCT device (QuantumFX, PerkinElmer, Waltham, MA, USA) using low-resolution scans (90 kV, 200 µA, field-of-view 20 × 20 mm^2^ and 10 × 10^2^ witm, respectively. The resulting density data were analyzed using a threshold-based algorithm (Scry 6.0, C. Dullin) (Figure 4) across a volume corresponding to the 7 × 5 mm cylindrical block volume with 0.1 mm allowance. The density range of newly formed bone was defined using density values of <7300 arbitrary units, and the volume was assessed in mm^3^. Pore volume fill was calculated from the technically defined pore volumes of 69 mm^3^ and 79 mm^3^, respectively.

For histologic preparation, the scaffolds with the surrounding bone were dehydrated and embedded in Technovit 9100© (Heraeus Kulzer GmbH, Philipp-Reis-Str. 8/13, 61273 Wehrheim, Germany). Thick section specimens were produced using a laser microtome (Tissue Surgeon, LLS ROWIAK, Hannover, Germany) (Line Overlap: 60%, Pulse Overlap: 77%, Pulse energy: 130 nJ, Index of Refraction: 1.5, Pulse Frequency: 10 MHz, Cutting depth: 50–55 µm) at a thickness of 40–45 µm perpendicular to the trephine defect axis. Approximately 10 to 12 specimens were produced from each implant and its surrounding bone perpendicular to the axis of the trephine cavity and divided between the following:

- Surface staining for histomorphometric assessment of bone formation using Alizarine red/Methylene Blue and van Gieson stains.
- Immunohistochemical staining of osteocalcin using peroxidase staining. For immunohistochemical staining, the specimens were mounted on Adhesive Microscope Slides (3800200AE, Leica Biosystems, Wetzlar, Germany) and deplastisized (according to ROWIAK, Hannover, Germany) by incubation in a mixture of xylene and MMA (1:1; Methyl methacrylate, Merck, Darmstadt, Germany) for 24 h, followed by incubation in xylene for 20 h. The specimens were then rehydrated in descending concentrations of ethanol (100%, 95%, 70%, for 5 min each) and washed in deionized water for 5 min. Deplasticized and rehydrated bone tissue sections were incubated in 1× citrate-based Target Retrieval Solution, pH 6.0 (Agilent Dako, Waldbronn, Germany) at 60 °C overnight. Afterwards, the sections were washed for 10 min in deionized water and three times in TBS for 5 min each, following treatment with 1 mL Trypsin Solution and 3 mL Trypsin Buffer (Trypsin Pretreatment Kit, Zytomed Systems, Berlin, Germany) in a humidity chamber at 37 °C for 20 min. The sections were washed for 5 min in deionized water and three times in TBS, 5 min each. To block endogenous peroxidase activity, the specimens were incubated with peroxidase-Blocking Solution (Dako, Waldbronn, Germany) in a humidity chamber at room temperature (RT) for 17 min and rinsed with TBS three times, 5 min each. Next, the samples were incubated in a humidity chamber for 1 h at RT in blocking buffer (10% goat Serum Block in PBS, Histoprime Biozol, Eching, Germany). Immunostaining was performed by incubation with a 1:50 dilution of BGLAP (Osteocalcin) monoclonal antibody (ABN-H00000632-M01, Abnova Biozol, Eching, Germany; humidity chamber, overnight at 4°C) followed by washing three times in TBS for 5 min each and incubation with an HRP-conjugated secondary antibody (Goat anti-Mouse, A10551, Thermo Fisher Scientific, Rockford, IL, USA; 1:250, humidity chamber, 1 h at RT). The antibodies were diluted using Antibody Diluent (Agilent Dako, Waldbrunn, Germany). After the samples were washed three times in TBS, 5 min each, the reactivity was detected using DAB (3,3’-Diaminobenzidine) substrate (Liquid DAB Substrate-Chromogen System, Agilent Dako, Waldbrunn, Germany; 20 min at RT). The reaction was stopped with deionized water, and the sections were rinsed four times in deionized water. Subsequently, specimens were counterstained with Mayer’s hemalaun solution (Merck, Darmstadt, Germany) for 4 s at RT, followed by incubation for 5 min in tap water at RT. The samples were dehydrated in ascending concentrations of ethanol (70% for 2 min, 96% twice for 2 min each, 100% twice for 5 min each) followed by clarification in xylene for 5 min. Finally, the specimens were mounted with Entellan (Merck, Darmstadt, Germany) and dried for 48 h at RT. Sections stained without the primary antibody served as controls.

For histomorphometric evaluation of bone formation, the specimens were scanned through a high-resolution camera (Axioskop 2 plus mit Axio Cam MRc5, Zeiss GmbH, Jena, Germany) at 1.25× magnification. For evaluation of immunohistochemistry, the specimens were scanned using a motor-driven table and a microscope camera (Zeiss Axiovert 200 M/AxioCam MRc Rev.3.FireWire, Zeiss, GmbH, Jena, Germany) at 10× magnification.

The resulting digital image data were analyzed using a custom-made Python3-(version 3.10.9) based image analysis pipeline (C.D.) utilizing the common Python modules scikit-image, matplotlib, opencv, and pandas.

**Figure 5:**
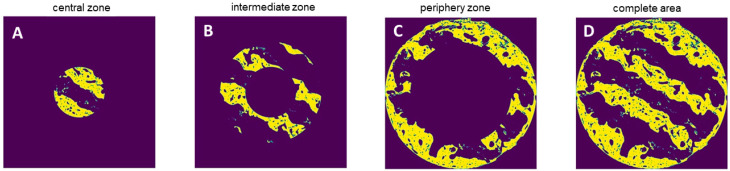
(**A**–**D**). Subsection analysis of the central, intermediate, and periphery zones and the entire cross-section area of the scaffolds. The areas of bone ingrowth identified by the automatic morphometry algorithm are highlighted in yellow.

For histomorphometry of both bone formation and osteocalcin expression, the algorithm automatically identified the color of the van Gieson/peroxidase-stained areas in the respective cross-section specimens and assessed the area occupied by bone and positive osteocalcin expression, respectively, in absolute values by pixel counting. Pixels were converted into mm^2^ using the calculated pixel size of 4.17 µm^2^/pixel for bone morphometry and 2.39 µm^2^/pixel for osteocalcin expression. The area of interest was defined as the circular cross-sectional area of the scaffolds. For subsection analysis, the area was divided into a central, intermediate, and periphery zone (Figure 5A–D).

### 2.6. Statistics

Results were reported as means and standard deviation. Results were tested for normal distribution using the Shapiro–Wilkes test for each of the variables. Only for 4 out of the 40 variables, a *p*-value < 0.05 was obtained, indicating that 90% of the variables had a normal distribution. Mean values of the area of newly formed bone and pore volume fill, and area of positive OC expression were compared between the four scaffolds using repeated measures ANOVA with Bonferroni correction for multiple testing in pairwise comparisons. Comparison between the 4 and 13 week intervals was performed using *t*-tests for unpaired samples (SPSS Statistics 24.0, http://support.spss.com). The significance level was defined as *p* < 0.05.

## 3. Results

As mentioned before, four out of the ten animals suffered a tibial fracture, resulting in six animals remaining in the experiment. These animals healed well without any further signs of complications.

### 3.1. Histology

**Figure 6:**
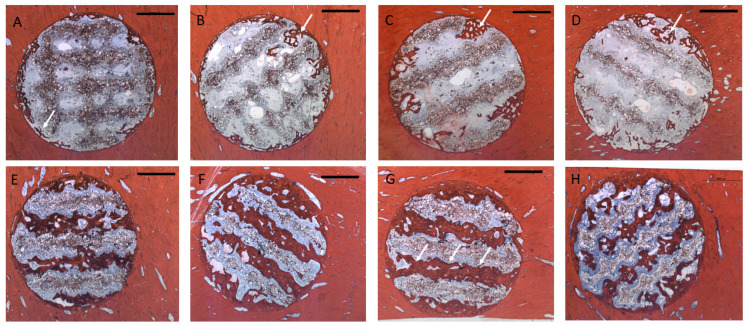
Representative cross-sections through the scaffolds, Alizarin–Methylene Blue stain (ar: 2000 µm): White arrows indicate bone ingrowth into the scaffolds from the defect walls. Upper row **4 weeks**: (**A**): Group 1/PLLA-CaCO_3_ scaffolds with 1000 µm scaffold web thickness (pore volume 69 mm^3^); (**B**): Group 2/PLLA-CaCO_3_ scaffolds with 800 µm scaffold web thickness, (pore volume 78 mm^3^); (**C**): Group 3/PLLA-CaCO_3_/CaP scaffolds with 1000 µm scaffold web thickness, (pore volume 69 mm^3^); (**D**): Group 4/PLLA-CaCO_3_/CaP scaffolds with 800 µm scaffold web thickness, (pore volume 78 mm^3^). Lower row: **13 weeks** (**E**): Group 1/PLLA-CaCO_3_ scaffolds with 1000 µm scaffold web thickness (pore volume 69 mm^3^); (**F**): Group 2/PLLA-CaCO_3_ scaffolds with 800 µm scaffold web thickness (pore volume 78 mm^3^); (**G**): Group 3/PLLA-CaCO_3_/CaP scaffolds with 1000 µm scaffold web thickness (pore volume 69 mm^3^). White arrows indicate bone ingrowth into the scaffolds with almost complete pore fill. (**H**): Group 4/PLLA-CaCO_3_/CaP scaffolds with 800 µm scaffold web thickness, (pore volume 78 mm^3^).

**Figure 7:**
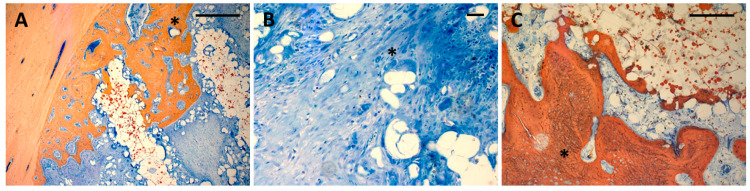
(**A**): Bone ingrowth into scaffolds from the defect wall after 4 weeks. Direct contact between bone and scaffold material, detached polymer particles in the pores of the scaffold. Some of the particles are incorporated into newly formed bone (asterisk). Ca containing particles can be seen from the red Alizarin stain inside the scaffold material (Bar: 250 µm); (**B**): Degradation of polymer particles through multinuclear giant cells (asterisk) (Bar 50 µm) (**C**): Bone formation in the central parts of the scaffold showing bone formation directly on the scaffold surface (Bar: 250 µm). Detached particles are no longer incorporated into the newly formed bone (asterisk) and are limited to the immediate vicinity of the scaffold remnants.

After *4 weeks*, bone formation extended 1–2 mm into the scaffold pores (Figure 6A–D). There was some degree of superficial disintegration of the scaffolds visible with polymer particles being detached from the scaffold surface (Figure 7A), undergoing degradation in the surrounding soft tissue (Figure 7B). There were also particles included in the ingrowing bone tissue. In many areas, the newly formed bone tissue was in immediate contact with the scaffold surface (Figure 7A). After *13 weeks*, bone tissue had almost completely penetrated the scaffold volume along the pore structure (Figure 6E–H). Most of the detached particles had been degraded, and bone tissue was in direct contact with the scaffold surface, with bone formed on the surface and inside the scaffold material between the polymer grains (Figure 7C). There was no obvious difference with respect to morphology or pattern of bone formation between the four different groups.

### 3.2. Immunohistochemistry

**Figure 8:**
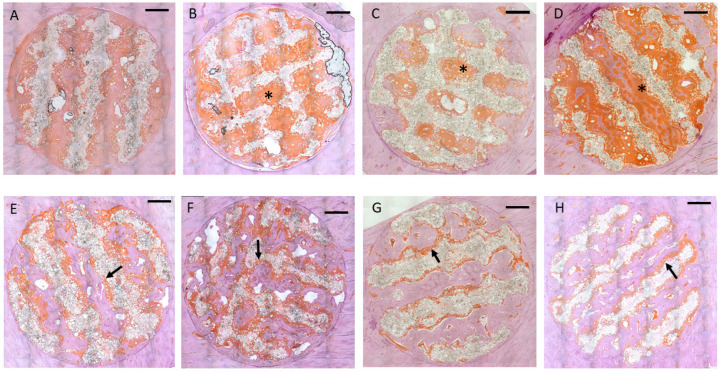
Representative cross-sections through the scaffolds, peroxidase stain of osteocalcin (Bar: 1000 µm): Intense positive reactions for osteocalcin throughout the tissue grown into the scaffold volume (black asterisks). Upper row **4 weeks**: (**A**): Group 1/PLLA-CaCO_3_ scaffolds with 1000 µm scaffold web thickness (pore volume 69 mm^3^); (**B**): Group 2/PLLA-CaCO_3_ scaffolds with 800 µm scaffold web thickness, (pore volume 78 mm^3^); (**C**): Group 3/PLLA-CaCO_3_/CaP scaffolds with 1000 µm scaffold web thickness, (pore volume 69 mm^3^); (**D**): Group 4/PLLA-CaCO_3_/CaP scaffolds with 800 µm scaffold web thickness, (pore volume 78 mm^3^). Lower Row: **13 weeks.** Positive reaction for osteocalcin has decreased to small seams on the surface of the newly formed bone and the scaffold surface (black arrows). (**E**): Group 1/PLLA-CaCO_3_ scaffolds with 1000 µm scaffold web thickness (pore volume 69 mm^3^); (**F**): Group 2/PLLA-CaCO_3_ scaffolds with 800 µm scaffold web thickness, (pore volume 78 mm^3^); (**G**): Group 3/PLLA-CaCO_3_/CaP scaffolds with 1000 µm scaffold web thickness, (pore volume 69 mm^3^); (**H**): Group 4/PLLA-CaCO_3_/CaP scaffolds with 800 µm scaffold web thickness, (pore volume 78 mm^3^).

**Figure 9:**
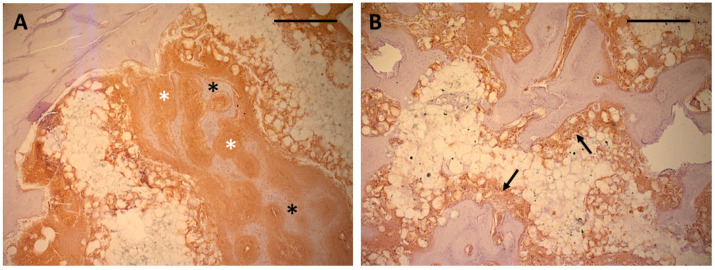
(**A**): Intense positive reaction for osteocalcin (white asterisk) in immature in-growing bone (black asterisk) after 4 weeks. Soft tissue invading the scaffold material has also shown a positive reaction in direct contact with the scaffold surface (bar: 500 µm). (**B**): Smaller seams of positively stained tissue between newly formed bone and scaffold surface in the center of the scaffolds after 13 weeks, indicating a continuing osteogenic differentiation also in immediate contact with the scaffold surface (black arrows). (Peroxidase stain of osteocalcin, bar: 500 µm.).

Immunohistochemical staining of osteocalcin showed extensive positive reaction evenly distributed throughout the entire soft tissue inside the scaffold after *4 weeks* (Figure 8A–D). Both the matrix adjacent to the immature bone and the soft tissue in immediate contact with the scaffold material exhibited intense staining of osteocalcin (Figure 9A). After 13 weeks, the areas of osteocalcin expression had substantially decreased, being limited to narrow seams of positively stained tissues on the surface of the newly formed bone (Figure 8E–H). Positive staining extended into the scaffold material between the polymer grains and around the detached polymer particles (Figure 9B).

### 3.3. Histomorphometry and Μct

**Figure 10:**
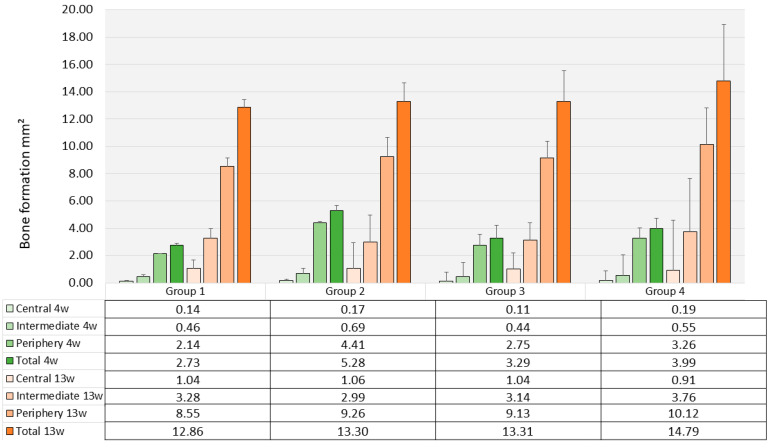
Bone formation inside the scaffolds (histomorphometry, mm^2^, mean values, and standard deviations, *n* = 3). Green bars indicate bone formation after 4 weeks; orange bars indicate bone formation after 13 weeks.

After *4 weeks*, the amount of newly formed bone varied considerably between a mean total area of 2.73 mm^2^ (SD 0.81) in Group 1 (PLLA-CaCO_3_, 69 mm^3^ pore volume) and 5.28 mm^2^ (SD 1.8) in Group 2 (PLLA-CaCO_3_, 79 mm^3^ pore volume) (Figure 10). Differences in mean values were not significant between the four types of scaffolds (*p* = 0.156). There was a clear decrease in bone formation from the periphery to the center of the scaffolds that was significant for all scaffold types (*p*-values varied from 0.002 to 0.019). Bone formation in the individual zones was not significantly different between the four types of scaffolds.

**Figure 11:**
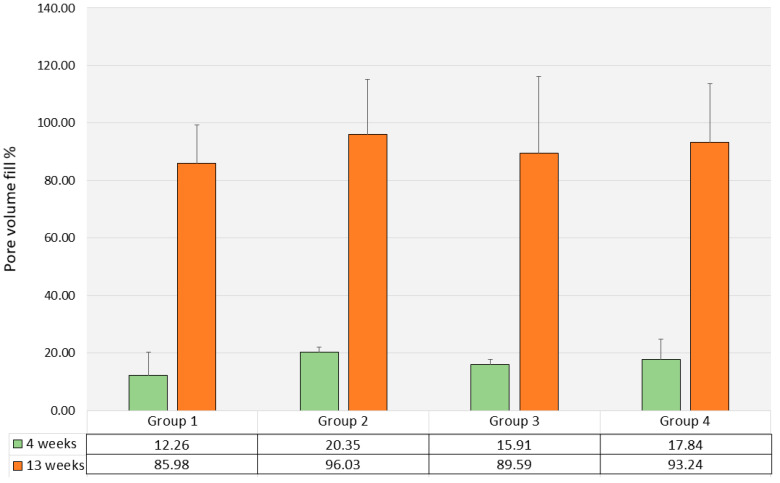
Pore volume fill (µCT, %, mean values and standard deviations, *n* = 3). Green bars indicate pore volume fill after 4 weeks; orange bars indicate pore volume fill after 13 weeks.

Mean pore volume fill as assessed by µCT varied between 12.25% (SD 9.71) in Group 1 and 20.35% (SD 1.92) in Group 2, without significant differences (*p* = 0.390) between the four groups (Figure 11).

**Figure 12:**
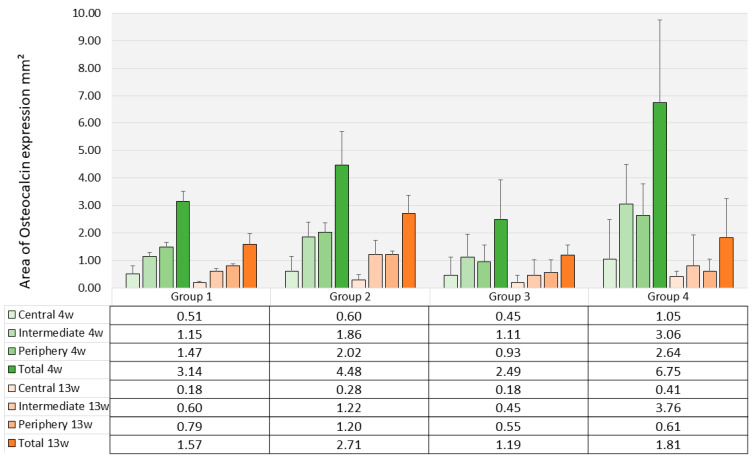
Osteocalcin expression inside the scaffolds (histomorphometry, mm^2^, mean values and standard deviations, *n* = 3). Green bars indicate area of osteocalcin expression after 4 weeks; orange bars indicate area of osteocalcin expression after 13 weeks.

The average level of osteocalcin expression as assessed through immunohistochemical staining ranged between 2.49 mm^2^ (SD 1.41) for Group 3 and 4.48 mm^2^ (SD 1.73) for Group 2 without significant differences between the groups (*p* = 0.126) (Figure 12).

Bone formation after *13 weeks* as assessed by histomorphometry had significantly increased in all scaffold groups (*p*-values ranging between 0.004 and 0.0.028). The highest amount of newly formed bone was found in Group 4 with a mean value of 14.79 mm^2^ (SD 5.06), and the lowest was registered in Group 1 (12.86 mm^2^, SD 4.50) (Figure 9). Again, differences in mean values between the groups were not significant (*p* = 0.361). There was a significant decrease in the amount of newly formed bone from the periphery towards the center of the scaffolds (*p*-values ranging from <0.001 to 0.002 for the four types of scaffolds). Bone formation in the individual zones was not significantly different between the four groups.

The mean pore volume fill had significantly increased from 4 weeks to 13 weeks (*p*-values ranging from 0.001 to 0.009) and varied between 86.0% (SD 16.24) in Group 1 and 96.0% (SD 23.36) in Group 2. Differences in mean pore volume fill between the different scaffold types were not significant (*p* = 0.479) (Figure 10).

The mean area with positive staining of osteocalcin after 4 weeks ranged between 1.18 mm^2^ for Group 3 (SD 0.53) and 2.71 mm^2^ (SD 1.39) for Group 2. Differences between the groups were not significant (*p* = 0.543). The decrease from 4 weeks to 13 weeks was not significant as well in the different scaffold types (*p*-values in the four groups varied between 0.052 and 0.120) (Figure 11).

## 4. Discussion

The present study has evaluated the in vivo performance of a resorbable PLA-bioceramic scaffold that had been produced through selective laser sintering (SLS). Most of the resorbable PLA-bioceramic scaffolds currently under research have been produced through extrusion-based techniques, where the heated polymer material is extruded through a computer-driven nozzle, such as fused deposition modeling (FDM) or other types of 3D printing, resulting in a three-dimensional structure of rectangular struts with a smooth surface [2]. The characteristics of PLA scaffolds produced through SLS from a powder bed are quite different depending on the type of PLA polymer and the laser energy employed. In vitro studies on SLS using poly-lactid-acid powder in conjunction with CaCO_3_ particles have shown [17] that poly-(L-lactide) is more suitable than poly-(DL-lactide) to produce open porous scaffolds due to superior sintering characteristics at the energy level used for the laser sintering process. The SLS-produced scaffolds are characterized by a rough inner surface with a certain degree of microporosity due to incompletely melted powder grains resulting from the fact that the process temperature does not lead to complete melting of the material [6,7]. This microporosity, in conjunction with bioceramic particles, is considered to facilitate cell attachment and capillary growth [8]. The surface features reported in previous studies compare well to the SEM findings and histologic results of the present study, showing unmelted polymer grains on the scaffold surface. The present study has shown that these unmelted particles detached in vivo during the first weeks of implantation and accumulated at a certain distance from the inner scaffold surface, where they underwent multinuclear giant cell degradation. This degradation process obviously did not interfere with bone ingrowth, as bone formation in many areas even occurred in direct contact with the PLLA-CaCO_3_/CaP scaffold surface, with mineralized tissue extending between the superficial polymer particles and incorporating individual particles into the ingrowing bone, as shown in the 4-week micrographs. As the present study is the first in vivo evaluation of this material, the exact fate of the detached particles remains unclear. However, the fact that detached particles undergo cellular degradation and are incorporated into ingrowing bone tissue after 4 weeks to the point where they are not seen anymore after 13 weeks suggests complete degradation of those particles that have migrated deeper into the pore volume.

The direct contact between bone and scaffold surface observed in the present study has also been reported for bone ingrowth into other polymer–bioceramic scaffolds (poly-vinyl-acid/hydroxyapatite) [8]. This is different from the features of bone ingrowth into pure poly-lactic acid scaffolds, where direct contact between newly formed bone and the scaffold surface has not been regularly observed [19]. The findings in the present study, thus, confirm previous findings that the incorporation of Ca-containing particles such as CaCO_3_ and CaP can enhance tissue integration of degradable polymers.

In the present study, bone ingrowth into the scaffolds appeared to originate from the surrounding bone, as shown by the significant decrease in bone formation towards the center of the scaffold in comparison to the scaffold periphery that is in contact with the pristine bone. This osteoconductive pattern of bone formation was seen after 4 weeks and even after 13 weeks in all types of scaffolds, indicating that bone regeneration in the central parts may not have been completed after that period. Immunohistochemical staining also showed that even after 13 months, the soft tissue in contact with the scaffold surface had maintained a state of osteogenic differentiation, indicating that osteogenesis can be expected to continue. This raises the question of whether the size of the defects was appropriate for a critical size defect and whether an empty control defect would have been required. The dimensions of cylindrical critical size bone defects reported in different skeletal sites of minipigs vary between 7.3 and 11 mm [26,27,28,29], indicating that the defect size chosen with 7 mm diameter in the current study could be considered as critical size defect. The rather high numbers of tibial fractures in the current experiment also suggest that an additional empty defect of this size would have been impossible to accommodate in the tibial metaphysis of the animals. As the approval of the veterinarian authority was limited to the use of one leg per animal, the omission of the control defect was the most preferable option considering the fact that the comparison of material characteristics was in focus of the experiment and the defect size compared well to the size of previously reported critical defects.

Quantitative assessment of bone ingrowth has shown that the amount of bone formed had significantly increased between 4 and 13 weeks in all four scaffold types. After 13 weeks, a high degree of pore volume fill was found, ranging between 86 and 96% corresponding to a bone volume fraction inside the whole scaffold between 30.9 and 39.5%. This volume fraction compares well to the newly formed bone volume between 11.2 and 39.9% reported for synthetic and block grafting materials in experimental applications in large animal models after up to 1 year [30]. When SLS produced resorbable polymer–bioceramic scaffolds are considered, the results of the present study are very much comparable to those previously reported for poly-caprolactone-hydroxyapatite scaffolds [6].

Comparison of bone formation between the different scaffold types at both intervals has shown that neither pore volume nor composition of the scaffolds had a significant effect on the amount of bone ingrowth. The same held true when the three individual zones inside the scaffolds were considered, indicating that the pattern of bone ingrowth was not affected by the differences, neither in pore volume nor composition, between the four scaffold types. The variation in composition by the surface modification of CaCO_3_ particles with CaP has been intended to enhance the bioactivity of the sintered surface. This approach is based on observations that the integration of hydroxyapatite nanoparticles into polymer fiber scaffolds has been shown to increase in vitro bone cell viability and activity as well as in vivo bone formation [31,32,33]. The micrographs in the present study have shown that the calcium-containing microparticles were evenly distributed across the polymer matrix of the scaffold. The quantitative results of bone formation inside the scaffolds and the micrographic features of the bone-to-scaffold contact suggest that with this type of particle presentation in the polymer matrix, the addition of CaP to the CaCO_3_ particles did not account for a difference in bone formation compared to the pure CaCO_3_ particles alone. A possible explanation for the lack of biological response to the added CaP particles may be that the added CaP was located exclusively on the surface of the CaCO_3_-spherulites that were embedded in the PLLA matrix. The exposure of CaP to cells attached to the scaffold surface was, therefore, most likely considerably lower than in the studies that had reported an increase in bioactivity and bone formation. In those studies, scaffold loading with CaP had been accomplished either by dissolving nano HA-particles in chloroform-dissolved PLA with subsequent scaffold printing or by soaking the scaffolds in simulated body fluid, leading to adsorption of nano hydroxylapatite crystals on the scaffold surface [31,32]. Those scaffolds are likely to have provided a more intense contact between ingrowing or attached cells with evenly distributed and easily available nano-HA on the surface of the scaffolds, thereby eliciting a stronger osteogenic response. Another reason may be that the biological response to the present composite structure of the CaCO_3_-sperolithes/PLLA matrix has already led to almost complete filling of the pores with bone tissue, leaving little room for significant improvement by the addition of CaP. Nevertheless, the increase in CaP content with thicker coating layers on the spherolithes may be a rewarding direction of future research to enhance the amount of bone formation in the modified.

## 5. Conclusions

The present study has shown that resorbable SLS produced PLLA-CaCO_3_/CaP with two different pore volumes and two different compositions allowed for bone ingrowth that was able to almost completely fill the provided pore volume after 13 weeks. In many areas, newly formed bone was in direct contact with the scaffold walls, and intervening soft tissue exhibited a terminal osteogenic differentiation as shown by the expression of osteocalcin. Differences in pore volume between the scaffolds did not account for significant differences in bone formation inside the scaffolds. The addition of CaP likewise did not lead to increased bone formation, most likely due to low availability of CaP to the biological environment.

## Data Availability

The data presented in this study are available upon request from the corresponding author.
